# Necrotizing Enterocolitis in Preterm Infants: An Inflammatory Condition at the Crossroads of Intestinal Immaturity, Dysbiosis, and Nutrition

**DOI:** 10.1155/ijpe/9191604

**Published:** 2026-02-15

**Authors:** Adonis Muganza Nyenga, Olivier Mukuku, Janet Sunguza Ziazia, Stanislas Okitotsho Wembonyama

**Affiliations:** ^1^ Department of Pediatrics, University of Lubumbashi, Lubumbashi, Democratic Republic of the Congo, unilu.ac.cd; ^2^ Department of Maternal and Child Health, Institut Supérieur des Techniques Médicales de Lubumbashi, Lubumbashi, Democratic Republic of the Congo, istmlubumbashi.net

**Keywords:** gut microbiota, necrotizing enterocolitis, neonatal nutrition, prematurity, preventive strategies

## Abstract

Necrotizing enterocolitis (NEC) remains one of the most devastating gastrointestinal emergencies in neonatology, primarily affecting preterm and low‐birthweight infants. Despite advances in neonatal intensive care that have markedly improved survival among these high‐risk populations, NEC continues to be associated with significant morbidity and mortality. It is a complex, multifactorial disease that develops at the crossroads of intestinal immaturity, gut microbiota dysbiosis, impaired mucosal immunity, and suboptimal nutritional practices—particularly the use of formula feeding versus breast milk. In high‐income countries, the increasing survival of extremely preterm infants has contributed to a higher incidence of NEC. Moreover, low‐ and middle‐income countries, including those in sub‐Saharan Africa, must anticipate a similar rise in NEC cases as neonatal care systems evolve and survival rates improve. Proactive strategies are therefore essential to mitigate the burden of this disease. This narrative literature review synthesizes current evidence on NEC pathogenesis, diagnostic approaches, key risk factors, and evidence‐based preventive interventions. Emphasis is placed on the role of enteral feeding practices, microbial colonization, and early identification of at‐risk neonates. Furthermore, the review explores strategic clinical practices such as the promotion of exclusive breastfeeding, the cautious use of antibiotics, and the potential role of probiotics. Ultimately, reducing NEC incidence and improving outcomes is crucial for lowering neonatal mortality rates globally, particularly in vulnerable preterm populations.

## 1. Introduction

Necrotizing enterocolitis (NEC) is the leading gastrointestinal (GI) emergency during the neonatal period, with a mortality rate approaching 30% [1, 2]. It represents a major cause of morbidity and mortality among preterm infants and those with low birthweights. Significant advances in neonatal intensive care have markedly improved the survival of premature newborns. However, this improved survival has concurrently led to an increased population at risk and a corresponding increase in the incidence of complications such as NEC [3].

Anatomically, NEC is defined by areas of intestinal necrosis of variable extent, which may involve both the ileum and the colon. It is characterized by hemorrhagic infarction of the intestinal wall, beginning at the luminal surface and potentially progressing to full‐thickness necrosis and perforation. It may initially present insidiously with feeding intolerance, which can rapidly progress to an acute abdomen, often accompanied by radiologic signs of pneumatosis intestinalis [4].

The incidence of NEC varies across time and geographical regions and is inversely proportional to gestational age. Several factors influence the variability in NEC incidence. In addition to methodological differences among studies, interregional variations in incidence may be explained by the wide variability of risk factors associated with NEC, most of which are modifiable. Therefore, with ongoing advances and improvements in the management of premature neonates, modifiable risk factors for NEC should be continuously reassessed.

As early as the 1980s, associations were reported between rapid increases in enteral feeding volumes and the onset of NEC [5]. Indeed, NEC cases are rarely observed before the initiation of enteral feeding and occur less frequently in breastfed neonates [6]. Since then, numerous other genetic and environmental factors have been identified as potential contributors. Nevertheless, the pathogenesis of NEC appears to be multifactorial, with intestinal immaturity, formula feeding, intestinal dysbiosis, and infection constituting key underlying mechanisms [7].

## 2. Epidemiology

Over the past four decades, significant concern has been raised regarding a possible substantial increase in the incidence of NEC due to improvements in the survival of extremely preterm infants and the management of very low–birthweight neonates [3, 8]. Conversely, recent data from certain regions reported improvements in NEC‐related morbidity and mortality attributed to the implementation of measures such as breastfeeding, antenatal corticosteroid therapy, delayed initiation of enteral feeding, and the use of prebiotics and probiotics [9–11].

The incidence of NEC is estimated at approximately 7% in neonatal intensive care units, with variability reaching 13% among very low–birthweight infants, depending on the setting. Although not statistically significant, differences have been reported between low‐income and high‐income regions. However, this comparison remains limited due to the scarcity of literature from low‐resource settings [5]. The variability in incidence may be attributed to three key factors: the at‐risk population, NEC risk factors, and genetic susceptibilities. The size of the at‐risk population is directly related to the quality of neonatal care, particularly advancements in resuscitation for extreme prematurity. The gestational age threshold for resuscitation is greater in low‐income countries than in high‐income countries, which explains the smaller at‐risk population in these regions [9]. Furthermore, regarding NEC risk factors, the variability in incidence suggests that the presence of modifiable risk factors is influenced by clinical practice habits. Clinicians should therefore focus on controlling these factors to standardize management and define effective preventive measures tailored to their local context.

The genetic basis for NEC is increasingly recognized and may explain the considerable variability in NEC risk according to the ethnic origin of populations. Epidemiological studies have fueled this suspicion of genetic predisposition. Indeed, the low incidence rates reported in certain regions, such as Japan, Switzerland, and Austria, contrast with the higher frequencies observed in North America, the United Kingdom, and Ireland. Similarly, intraregional variability has been reported among ethnic groups, notably in the United States, where the risk is greater in African American populations than in Caucasians [12–14].

Accordingly, numerous studies have reported the influence of genetics on both the risk and severity of NEC. The authors highlighted that single‐nucleotide polymorphisms (SNPs) in various genes may play a crucial role in this genetic susceptibility. As the most common and extensively studied type of genetic variation, SNPs have been particularly implicated in modulating NEC risk [15, 16].

## 3. Pathogenesis and Risk Factors

NEC is a multifactorial condition, and several aspects of its pathogenesis are still not fully understood. Numerous risk factors have been reported in the literature, although expert opinions often diverge in terms of their relative importance.

**Figure 1:**
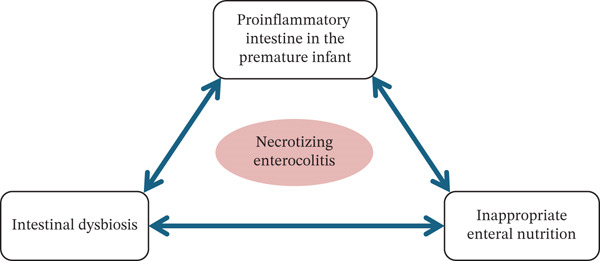
Determinants of necrotizing enterocolitis development.

To date, susceptibility to NEC is primarily determined by both functional and structural immaturity of the premature infant′s intestine, abnormal development of the intestinal microbiota (dysbiosis), and the nature of enteral feeding—particularly milk‐based formulas (Figure 1) [17, 18].

**Figure 2:**
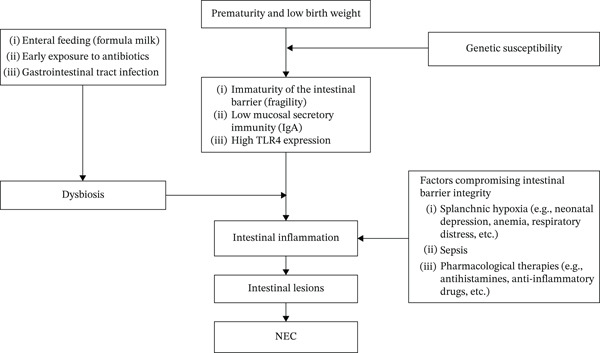
Pathogenesis and risk factors for NEC.

The pathogenesis of NEC primarily involves the convergence of three key elements: colonization of the intestine by inappropriate microorganisms, the presence of enteral nutrition within the intestinal lumen, and a triggering event that compromises the integrity of an already immature mucosal barrier. The development of NEC results from a complex interaction between the type of enteral feeding, the emergence of dysbiosis, and an exaggerated inflammatory response at the level of the intestinal mucosa (Figure 2) [19].

Several experimental studies have demonstrated the existence of a proinflammatory response in the intestinal mucosa of preterm infants, driven by the interaction between bacterial signalling receptors in the gut and an abnormal intestinal microbiota. The expression of the intestinal lipopolysaccharide receptor Toll‐like receptor 4 (TLR4) is reportedly higher in preterm neonates than in term infants. TLR4 is believed to play a critical role in regulating normal intestinal development, which may explain its elevated expression before full‐term gestation [1, 20].

### 3.1. Prematurity

Although NEC can also occur in term neonates, preterm infants—particularly those with very low birthweights—are universally recognized as being at the highest risk for NEC. The structural and functional immaturity of the GI tract in preterm infants makes them particularly vulnerable to various forms of intestinal injury. The preterm gut is characterized by underdeveloped intestinal motility, immature regulation of splanchnic circulation, and insufficient development of both the intestinal barrier and mucosal immune defenses. Key protective factors such as gastric acidity, digestive enzymes, intestinal mucus production, and secretory immunoglobulin A (IgA) play crucial roles in defending the GI tract against a wide range of insults. However, in preterm infants, these protective mechanisms are generally deficient [21]. As a result, additional factors—such as splanchnic hypoxia, enteral feeding with formula, and exposure to antibiotics—can further compromise the fragile mucosal barrier of the premature intestine [4, 22].

Furthermore, the functional immaturity of the immune system in preterm infants is implicated in the pathogenesis of NEC. The intestinal epithelium of preterm neonates exhibits excessive proinflammatory activity, primarily due to the increased expression of TLR4. This proinflammatory profile of the premature gut is now considered a central element in the mucosal necrosis process. Consequently, a massive and uncontrolled inflammatory response within the intestine is believed to lead to epithelial cell necrosis [23–25].

### 3.2. Intestinal Dysbiosis

The role of intestinal dysbiosis in the pathogenesis of NEC is widely acknowledged and supported by consensus. Several studies have reported the presence of dysbiosis days before the onset of clinical signs of NEC. The gut microbiome of infants who develop NEC is typically characterized by low microbial diversity, with a predominance of Proteobacteria at the expense of Firmicutes and Bacteroidetes [26–28]. The establishment of the intestinal microbiota is crucial for the proper functioning and integrity of the GI tract. From birth, the neonate is exposed to environmental bacteria—ranging from the maternal vaginal flora to microbes introduced through enteral feeding. These initial colonizing bacteria play a critical role in shaping the infant microbiome. The patterns of intestinal colonization help modulate the mucosal inflammatory response via TLR4. Factors specific to prematurity and its medical management can delay the development of a healthy microbiome, often resulting in an abnormal microbial profile (dysbiosis). This dysbiosis predisposes neonates to dysregulated inflammatory responses and altered bacterial glycosylation processes [29].

Clinical practices such as early exposure to broad‐spectrum antibiotics, delayed initiation of breastfeeding, and feeding with formula products contribute to delayed and abnormal microbial colonization. This dysbiotic state compromises the integrity of the intestinal mucosal barrier, increasing its susceptibility to infections.

### 3.3. Other Risk Factors

Several additional factors related to fetal adaptation to extrauterine life and early neonatal care have been reported in the literature. These include hemodynamic disturbances—such as hypoperfusion, hypovolemia, and/or anemia—often assessed through the Apgar score at 5 min of life. A significant association has been established between neonatal depression (low Apgar score) and an increased risk of developing NEC [17, 30].

Any factor that contributes to hypoxic events in low‐birthweight neonates plays a crucial role in NEC development. Accordingly, conditions such as respiratory distress and recurrent apnea, along with therapeutic interventions such as mechanical ventilation and umbilical catheterization, are significantly associated with the onset of NEC.

Feeding with infant formula and certain pharmacological treatments—including H2 receptor antagonists, indomethacin, and glucocorticoids—are recognized as predisposing factors for NEC. The hyperosmolar nature of infant formulas and other orally administered substances (e.g., medications with hyperosmolar excipients) is implicated in causing ischemic injury to the intestinal mucosa.

Last, rarer factors such as congenital malformations—especially those affecting the cardiovascular system and GI tract—may directly or indirectly increase the risk of NEC. Various other modifiable risk factors related to environmental and clinical conditions specific to each region have also been reported and remain under investigation [8, 21, 31].

## 4. Clinical Manifestations

The clinical presentation of NEC is variable, ranging from insidious forms with spontaneous resolution to severe forms with fulminant progression. Early signs are generally nonspecific and may resemble neonatal sepsis; however, the presence of concurrent GI symptoms points toward NEC. Nonspecific systemic signs such as lethargy, severe bradypnea, bradycardia, and temperature instability may represent early manifestations of a subtly evolving disease. Clinical suspicion is based on signs of feeding intolerance upon the initiation of enteral nutrition. These symptoms include tender abdominal distension accompanied by signs of discomfort and persistent gastric residual vomiting that quickly becomes bilious. This can rapidly progress to subocclusive abdominal syndrome with diarrheal stools, sometimes containing blood. Abdominal distension may become pronounced, with an edematous, shiny abdominal wall revealing collateral circulation [32, 33]. The general condition deteriorates rapidly, with clinical progression toward peritonitis, sepsis complicated by acidosis, coagulation disorders, and, ultimately, a state of shock. A differential diagnosis must be made with spontaneous intestinal perforation, which can occur before the initiation of enteral feeding. Unlike NEC, spontaneous intestinal perforation is typically localized and characterized by isolated mucosal ulceration, with the surrounding tissues appearing normal and a thinning of the submucosa. Although the clinical presentation of both conditions shares many similarities, the consistent appearance of a bluish discolouration of the abdomen in spontaneous intestinal perforation serves as an important distinguishing feature. Moreover, consideration of the risk factors associated with the development of NEC can help guide the diagnostic process [34, 35]. Several other clinical entities may mimic NEC, including ischemic intestinal necrosis, congenital intestinal anomalies that resemble NEC, and food protein‐induced enterocolitis syndrome [35].

Early diagnosis is crucial for reducing the morbidity and mortality associated with the disease and for improving patient prognosis. To this end, Bell′s criteria (Table 1) were developed to provide a clinical staging system for NEC, initially proposed by Bell et al. in 1978 [37] and later modified by Walsh and Kliegman in 1986 [36].

**Table 1:** Modified Bell′s criteria [36].

Stage	Systemic signs	Abdominal signs	Radiographic signs	Management
Ia: suspected NEC	—Temperature instability, apnea, bradycardia, lethargy	—Gastric residuals, abdominal distension, vomiting, hematochezia	—Normal or mildly dilated bowel loops, mild ileus	—Nil per os + antibiotics for 3 days—awaiting culture results
Ib: suspected NEC	—Temperature instability, apnea, bradycardia, lethargy	—Gastric residuals, abdominal distension, vomiting, hematochezia—rectal bleeding	—Normal or mildly dilated bowel loops, mild ileus	—Nil per os + antibiotics for 3 days—awaiting culture results
IIa: confirmed NEC (mild)	—Temperature instability, apnea, bradycardia, lethargy	—Gastric residuals, abdominal distension, vomiting, hematochezia—rectal bleeding—absent bowel sounds, with or without tenderness	—Bowel dilatation, ileus —pneumatosis intestinalis	—Nil per os + antibiotics for 7–10 days if exam is normal in 24–48 h
IIb: confirmed NEC (moderate)	—Temperature instability, apnea, bradycardia, lethargy—mild metabolic acidosis, thrombocytopenia	—Gastric residuals, abdominal distension, vomiting, hematochezia—rectal bleeding—absent bowel sounds, with or without tenderness, with or without abdominal cellulitis or right lower quadrant mass	—Bowel dilatation, ileus—pneumatosis intestinalis with or without ascites and portal venous gas	—Nil per os + antibiotics for 14 days—NaHCO_3_ for acidosis
IIIa: advanced NEC (severe with intact bowel)	—Temperature instability, severe apneas, bradycardia, lethargy—hypotension, mixed acidosis, disseminated intravascular coagulation (DIC), neutropenia	—Gastric residuals, abdominal distension, vomiting, hematochezia—rectal bleeding—absent bowel sounds, with or without tenderness, with or without abdominal cellulitis or right lower quadrant mass—signs of peritonitis, marked tenderness and distension	—Bowel dilatation, ileus—pneumatosis intestinalis with or without ascites and portal venous gas	—Nil per os + antibiotics for 14 days—NaHCO_3_ for acidosis—fluid resuscitation (200 mL/kg)—Inotropic support—oxygen therapy—paracentesis
IIIb: advanced NEC (with intestinal perforation)	—Temperature instability, severe apneas, bradycardia, lethargy—hypotension, mixed acidosis, DIC, neutropenia	—Gastric residuals, abdominal distension, vomiting, hematochezia—rectal bleeding—absent bowel sounds, with or without tenderness, with or without abdominal cellulitis or right lower quadrant mass—signs of peritonitis, marked tenderness and distension	—Bowel dilatation, ileus—pneumatosis intestinalis with or without ascites and portal venous gas, with pneumoperitoneum	—Nil per os + antibiotics for 14 days—NaHCO_3_ for acidosis—fluid resuscitation (200 mL/kg)—inotropic support—oxygen therapy—paracentesis —surgical intervention

## 5. Diagnosis

Standard abdominal radiography and intestinal ultrasound constitute the cornerstone of NEC diagnosis [18, 38, 39]. Depending on the stage of the disease, radiographic findings range from signs of pneumatosis intestinalis (presence of intramural gas) or portal venous gas (Bell Stages IIa to IIIa) to clear evidence of pneumoperitoneum, which indicates intestinal perforation (Bell Stage IIIb). Although these signs are specific, they are not consistently present on all radiographs. Intermediate findings such as gaseous bowel distension, air–fluid levels, bowel wall thickening, and ascites are common and not uncommon. In addition to being noninvasive and capable of detecting the classic signs of NEC (pneumatosis intestinalis, portal venous gas, and pneumoperitoneum), intestinal ultrasound allows for the measurement of bowel wall thickness and the assessment of peristalsis and intestinal perfusion via color Doppler. Moreover, it enables better characterization of ascites. These capabilities make intestinal ultrasound superior to plain radiography for early diagnostic approaches (Bell Stage I) and for detecting insidious clinical progression in high‐risk neonates [38, 40].

Although plain radiography remains the gold standard for NEC diagnosis, the advantages of intestinal ultrasound suggest that a combined approach using both modalities may enhance the prediction of the need for surgical intervention [40, 41].

More recently, near‐infrared spectroscopy (NIRS) has emerged as a promising noninvasive adjunct for early detection of splanchnic hypoxia in preterm infants [42–44]. NIRS is a noninvasive technique that uses light from the near‐infrared spectrum to assess regional tissue oxygenation. Reduced splanchnic oxygenation measured by NIRS has been associated with the development and severity of NEC, potentially enabling earlier risk stratification and continuous bedside monitoring [42]. However, substantial heterogeneity in the predictive value of NIRS measures in ECUN has been noted between different studies. Thus, its routine clinical use remains investigational and requires further validation before widespread implementation [42, 45].

A number of additional molecular markers have been investigated to improve the early diagnosis of NEC in preterm infants. These include acute‐phase inflammatory markers such as C‐reactive protein (CRP) and serum amyloid A (SAA), proinflammatory cytokines (TNF‐*α*, IL‐6, and IL‐8), and organ‐specific biomarkers indicative of enterocyte injury or compromised intestinal barrier integrity. Notably, these include fecal calprotectin, claudin‐3, trefoil factor 3, intestinal fatty acid–binding protein (I‐FABP), and liver fatty acid–binding protein (L‐FABP) [40].

Urinary levels of I‐FABP have shown promise in predicting NEC and assessing the extent of intestinal necrosis. This protein, which is involved in enterocyte lipid metabolism, is released into the circulation following enterocyte injury. The value of these biomarkers in the early identification of NEC remains limited by their restricted availability in routine clinical practice. As a result, many clinicians continue to rely on Bell′s criteria for the diagnosis and management of NEC [39, 46].

## 6. Clinical Strategies for NEC Prevention

In addition to our understanding of the pathogenesis and risk factors, several clinical strategies have proven effective in the prevention of NEC. These include the use of human breast milk, minimizing exposure to antibiotics, probiotic supplementation, and the implementation of standardized protocols for the initiation of enteral feeding in at‐risk neonates [7, 47].

### 6.1. Use of Human Breast Milk

One of the well‐documented properties of human breast milk is its ability to increase host defenses and support GI function in newborns. Its dynamic array of bioactive components is essential for neonatal growth and the maturation of the intestinal and immune systems. Several studies have confirmed the protective role of human milk in the prevention of NEC [17, 19, 30, 48–52].

The benefit of human milk lies largely in the role of its oligosaccharides and microbiome in shaping favorable and specific neonatal gut microbiota. Numerous studies have reported the efficacy of human milk oligosaccharides (HMOs) in preventing NEC and have even suggested HMO supplementation in nutritional strategies for low‐birthweight neonates [50, 51]. Furthermore, the presence of immunomodulatory metabolites and growth factors plays a critical role. Nolan et al. [52] demonstrated the protective effects of secretory IgA and growth factors present in human milk against NEC. These components enhance the immune defenses of neonates and modulate the inflammatory response responsible for necrotic lesions, notably through the inhibition of TLR4 signalling [20]. The use of human breast milk in place of formula is therefore a cornerstone of NEC prevention in routine clinical practice.

### 6.2. Probiotic Supplementation

For over a decade, the intestinal microbiota has been recognized as a key factor in both health and disease among preterm infants [53]. Multiple factors can significantly disrupt the neonatal gut microbiota, including mode of delivery, exposure to antimicrobials, and the nature of the infant′s diet (e.g., formula feeding). In general, probiotic supplementation is aimed at preventing dysbiosis and promote nutrient metabolism through improved gut health. Numerous studies have demonstrated the beneficial effects of probiotics in preventing NEC and their positive influence on the growth and development of preterm infants [54–58]. Evidence from randomized controlled trials and meta‐analyses indicates that probiotic supplementation reduces the incidence of NEC, late‐onset sepsis, and all‐cause mortality in very preterm infants [59–64]. The most consistently beneficial strains include *Bifidobacterium* species (particularly *Bifidobacterium breve* and *Bifidobacterium infantis*), *Lactobacillus* species (notably *Lactobacillus rhamnosus* GG), and multistrain or multispecies preparations combining *Bifidobacterium* and *Lactobacillus*. The benefit of using multistrain or multispecies probiotics compared with single‐strain probiotics remains controversial, although the hypothesis of a potential synergistic effect resulting from the combination of the probiotic effects of different species is raised [63, 65, 66]. These probiotics are thought to enhance intestinal barrier maturation, modulate inflammatory responses, and restore microbial balance. However, strain specificity, product quality, and regulatory oversight remain critical considerations, especially in vulnerable neonatal populations [63, 65].

### 6.3. Standardized Nutritional Approach

A link has been reported between enteral feeding and the occurrence of NEC. One of the iatrogenic components contributing to NEC development is believed to be associated with the type of feeding regimen and nonstandardized nutritional practices. As such, the concept of a standardized feeding protocol has become integrated into clinical practices aimed at preventing NEC in at‐risk neonates. This involves not only the appropriate selection of the feeding source (i.e., human milk) but also the early and codified initiation of enteral nutrition.

The protective effect of breast milk in preventing NEC is well established. Owing to its capacity to attenuate the proinflammatory response mediated by TLR4 and its immunological role in preventing dysbiosis—through the action of secretory IgA, lactoferrin, HMOs, and growth factors—maternal milk or donor human milk remains the recommended choice within a standardized nutritional approach for neonates at risk of NEC [67]. Standardized nutritional protocols encourage early initiation of enteral feeding with human milk on the basis of evidence that this reduces the risk of feeding intolerance and allows for the early detection of warning signs that may prompt additional interventions to minimize the risk of NEC [68]. There has been longstanding debate regarding the benefits of slow advancement of enteral feeding volumes. However, a recent meta‐analysis concluded that slow advancement of feeding volumes likely does not impact the risk of NEC [69]. Similarly, Salas et al. [70], Thoene et al. [71], and Dorling et al. [72] reported that early introduction and rapid advancement of enteral feeding volumes are feasible—even in very low–birthweight infants—without increasing the risk of NEC. This “aggressive” feeding strategy is instead associated with a shorter duration of central catheter use, reduced reliance on parenteral nutrition, and improved weight gain.

## 7. Management

Upon clinical suspicion of NEC, a medical approach is immediately recommended. This typically involves cessation of enteral feeding (GI rest), gastric decompression via nasogastric tube placement, systemic antibiotic therapy, and initiation of parenteral nutrition [35, 73]. Broad‐spectrum antibiotic regimens are generally employed, although the choice of agents should be guided by local antimicrobial resistance patterns. Several regimens have been proposed, with a treatment duration of 7–14 days considered optimal [33, 74].

Depending on the clinical stage of the disease, additional supportive and resuscitative measures may be necessary. These include maintaining circulatory stability, correcting metabolic acidosis, and addressing hematological abnormalities, particularly coagulopathy and anemia [21].

Close clinical and paraclinical monitoring is crucial to determine the need for surgical intervention. The presence of pneumoperitoneum constitutes an absolute indication for surgery. In the absence of pneumoperitoneum, relative indications for surgical intervention are more nuanced and rely primarily on the degree of clinical deterioration despite medical management. Findings such as abdominal wall edema or discoloration, persistent hemodynamic instability, worsening acidosis or thrombocytopenia, and radiographic signs (including extensive pneumatosis or portal venous gas) may support the decision to operate. Timely surgical decision‐making has a significant effect on patient prognosis [34]. Outcomes tend to be poorer when surgery is considered only after medical treatment has failed. Ideally, surgery should be performed after irreversible intestinal necrosis has occurred but before perforation—although predicting this precise moment is challenging. Early surgical intervention thus presents a potential opportunity to improve patient outcomes. Surgical management primarily involves exploratory laparotomy with resection of necrotic bowel, followed by primary anastomosis or, more commonly, stoma formation depending on the extent of disease and the infant′s clinical stability [75, 76]. Peritoneal drainage may be used as a temporizing measure, particularly in extremely low birthweight (< 1000 g) or hemodynamically unstable infants, either as a bridge to definitive surgery or, in selected cases, as sole intervention [77, 78]. Surgical NEC is associated with substantial morbidity and mortality, as well as long‐term complications such as short bowel syndrome and neurodevelopmental impairment, underscoring the importance of early recognition, prevention, and timely intervention [77, 79].

## 8. NEC in Low‐Resource Settings

Limited data are available on NEC in developing regions, particularly in sub‐Saharan Africa. Although these regions have a lower proportion of at‐risk populations for NEC, the condition is nonetheless associated with a high mortality rate in such settings [80].

The challenges encountered in the management of NEC are multifaceted. Beyond limited access to diagnostic tools, there are significant difficulties in implementing clinical prevention strategies. Several concerning issues include the following:

• the lack of a formal human milk donation policy,
• the widespread and often systematic use of broad‐spectrum antibiotics in extremely preterm neonates during the early neonatal period,
• the absence of probiotic supplementation in standard treatment protocols, and
• the unavailability of parenteral nutrition products.

Compared with the rest of the world, developing countries face considerable delays in initiating and sustaining human milk banking services. In many developing regions, human milk banks are simply nonexistent [81, 82]. In addition to infrastructure and equipment limitations, cultural beliefs act as major barriers to the organization of human milk donations. For example, in sub‐Saharan Africa, cultural norms often render the concept of feeding a child with milk from another woman unacceptable [83, 84]. In cases where the mother dies, the use of commercial infant formula is almost systematic—unless a close relative who is lactating agrees to adopt and breastfeed the orphaned newborn.

The overuse of antibiotics during the neonatal period, particularly in preterm infants, represents a major challenge in developing countries. In contexts where diagnostic tools for infection are inaccessible and clinical monitoring is limited, extreme prematurity is often perceived as a major risk factor for infection. Consequently, owing to the lack of strict adherence to standard aseptic measures, broad‐spectrum antibiotic therapy is typically initiated at birth in preterm infants [85–88]. This practice disrupts the establishment of a healthy gut microbiome, which is crucial for the digestive health of preterm infants.

Late diagnosis of NEC often leads to delayed management, resulting in a poor prognosis. The unavailability of parenteral nutrition products means that, in most cases, NEC is accompanied by severe malnutrition. This not only complicates surgical decision‐making but also worsens postoperative outcomes [89, 90].

## 9. Conclusion

NEC is one of the most critical GI emergencies in premature newborns. It is associated with significant morbidity and mortality. Although the complexity of its pathogenesis is still under investigation, the proinflammatory nature of the immature preterm intestine forms the physiological basis of the disease. Although progress has been made in identifying risk factors, many developing regions remain limited in their capacity to implement clinical prevention strategies and access diagnostic tools. Early diagnosis is essential for a favorable prognosis. Careful clinical and paraclinical monitoring allows timely and appropriate surgical intervention.

**Nomenclature**
CRP: C‐reactive protein
NEC: necrotizing enterocolitis
I‐FABP: intestinal fatty acid–binding protein
IgA: immunoglobulin A
IL‐6: interleukin 6
IL‐8: interleukin 8
SAA: serum amyloid A
SNPs: single‐nucleotide polymorphisms
TLR4: Toll‐like receptor 4
TNF‐*α*
: tumor necrosis factor alpha

## Author Contributions

A.M.N., O.M., and S.O.W. conceptualized the study and designed the methodology; A.M.N., O.M., and J.S.Z. conducted the literature review, critical review of the manuscript, and refinement of scientific content; A.M.N., O.M., and J.S.Z. prepared the original draft of the manuscript; S.O.W. provided senior supervision and contributed to the critical review and editing.

## Funding

No funding was received for this manuscript.

## Disclosure

All authors reviewed and approved the final version of the manuscript for publication.

## Ethics Statement

The authors have nothing to report.

## Conflicts of Interest

The authors declare no conflicts of interest.

## Data Availability

Data sharing is not applicable to this article as no datasets were generated or analyzed during the current study.
